# Slicc damage index score in systemic lupus erythematosus patients and its associated factors: Erratum

**DOI:** 10.1097/MD.0000000000013756

**Published:** 2018-12-14

In the article, “Slicc damage index score in systemic lupus erythematosus patients and its associated factors”,^[1]^ which appeared in Volume 97, Issue 42 of *Medicine*, the title should appear as “SLICC damage index score in systemic lupus erythematosus patients and its associated factors.”
